# Early Actions of Neurotransmitters During Cortex Development and Maturation of Reprogrammed Neurons

**DOI:** 10.3389/fnsyn.2019.00033

**Published:** 2019-11-21

**Authors:** Jorge Ojeda, Ariel Ávila

**Affiliations:** Developmental Neurobiology Unit, Biomedical Sciences Research Laboratory, Basic Sciences Department, Faculty of Medicine, Universidad Católica de la Santísima Concepción, Concepción, Chile

**Keywords:** neurotransmitters, hiPSC derived neurons, cortex, GABA, glycine, glutamate, neurogenesis, neurodevelopmental disorder

## Abstract

The development of the brain is shaped by a myriad of factors among which neurotransmitters play remarkable roles before and during the formation and maturation of synaptic circuits. Cellular processes such as neurogenesis, morphological development, synaptogenesis and maturation of synapses are temporary and spatially regulated by the local or distal influence of neurotransmitters in the developing cortex. Thus, research on this area has contributed to the understanding of fundamental mechanisms of brain development and to shed light on the etiology of various human neurodevelopmental disorders such as autism and Rett syndrome (RTT), among others. Recently, the field of neuroscience has been shaken by an explosive advance of experimental approaches linked to the use of induced pluripotent stem cells and reprogrammed neurons. This new technology has allowed researchers for the first time to model in the lab the unique events that take place during early human brain development and to explore the mechanisms that cause synaptopathies. In this context, the role of neurotransmitters during early stages of cortex development is beginning to be re-evaluated and a revision of the state of the art has become necessary in a time when new protocols are being worked out to differentiate stem cells into functional neurons. New perspectives on reconsidering the function of neurotransmitters include opportunities for methodological advances, a better understanding of the origin of mental disorders and the potential for development of new treatments.

## Introduction

The development of the brain cortex is an evolutionarily conserved process that starts during early embryonic stages and continues throughout childhood and adolescent all the way to adulthood (Kast and Levitt, 2019). In such a long process, there are plenty of events in which the developmental path can be altered and thus, the factors that influence the early stages of brain development can have profound effects on the functioning of an otherwise normal brain. In this context, neurotransmitters are among the most important extracellular cues that control the development of the cortex. Remarkably, these classical communication molecules have been shown to participate in a range of cellular processes that include cellular division, differentiation, neurogenesis, migration, morphological development, synapse formation, synaptic pruning and circuit maturation.

Neurotransmitters are fundamental factors involved in neuronal communication. They are well known and continue to be studied for their role at synapses mediating the transmission of information throughout the entire nervous system. In this regard, undoubtedly synapses are central for the functioning of the brain. Thus, the last 40 years have seen a tremendous advance in our understanding of the role of neurotransmitters at the synapses (Snyder, 2009). However, neurotransmitters are, now for about two decades, beginning to be recognized as pleiotropic extracellular signaling molecules with roles that span far beyond synaptic communication (Ascenzi and Bony, 2017). More in detail, neurotransmitters can act on ionotropic or metabotropic receptors, being the response mediated by the ionotropic receptors in the order of milliseconds and more than 10-times faster compared to metabotropic receptors. This difference in the speed of action relates to their intrinsic properties. While ionotropic actions are based on the flux of ions through the membrane and the subsequent change on resting membrane potential (RMP), metabotropic actions are mediated by the activation of G proteins. Nevertheless, the activation of ionotropic channels can also lead to the activation of downstream signaling cascades that merge onto G protein-mediated signaling. This is particularly relevant during development when ionic gradients are being established and the actions of ionotropic receptors are pleiotropic.

Here, we discuss the role of the most relevant neurotransmitters γ-aminobutyric acid (GABA, glycine and glutamate) focused on the neurogenesis, and synapse development including what begins to be discovered using reprogrammed neurons. Moreover, to keep the length of this review to a reasonable extent and to appropriately cover the recent discoveries, we will focus on ionotropic receptors, the developing cerebral cortex and the models that are currently being put forward to understand this system.

## Non-synaptic Functions of Neurotransmitters During Brain Development

Early experiments demonstrated that knocking out synaptic release machinery did not interfere with normal embryonic brain development in terms of axon targeting, layering and generation of gross morphological features (Verhage et al., 2000). However, before synapse formation, neurotransmitters are released by non-vesicular transport or through a process not requiring SNARE machinery (Demarque et al., 2002). Thus, although independently of vesicular release, we now know that neurotransmitters can have important effects in the structuring of the brain cortex.

### GABA_A_ Receptors

The GABA is arguably the most important neurotransmitter acting during embryonic development prior to synapse formation. Early experiments demonstrated that GABA was able to tonically activate neurons before synapse formation (Demarque et al., 2002). In line with this, early on it was found that GABA was able to inhibit DNA synthesis through the activation of GABA_A_ receptors (GABA_A_; LoTurco et al., 1995). More recently, similar findings have been described on retinal progenitors where more detailed analyses have linked the effects of GABA to the increase of intracellular calcium (Wang et al., 2019), which may as well be involved in the activation of CREB signaling (Jagasia et al., 2009).

Characteristically, both excitatory and inhibitory neurons of the cortex are born away from their final place having to migrate from the ventricular and subventricular zones where they are generated. For excitatory neurons, this means radial migration while for inhibitory neurons this involves a long road ahead in a process named tangential migration. In this context, GABA receptor activation was first found to exert a chemotropic effect on main cortical neurons (Behar et al., 1996). Additionally, it has been shown that activation of GABA_A_ regulates radial migration at birth by affecting the dynamics of intracellular calcium of migrating neurons (Wang et al., 2003; Heck et al., 2007). Likewise, activation of GABA_A_ at the rostral migratory stream during early postnatal development has been shown to modulate cell migration leading to the formation of the Islands of Calleja (Hsieh and Puche, 2015). Using the overexpression of a sodium channel as a tool to increase excitability and the frequency of calcium transients, it was found that precocious increase of calcium spikes induced migration arrest and the generation of neural processes (Bando et al., 2016). In line with that, a complementary approach increasing the expression of the potassium chloride transporter KCC2, the major extruder of Cl^−^ ions in mature neurons, leads to arrested morphological development of pyramidal neurons through the blockage of the excitatory action of GABA during embryonic development of the cortex (Cancedda et al., 2007). A similar role and mechanism of action involving GABA_A_ activation was described on migratory interneurons (Cuzon et al., 2006; Bortone and Polleux, 2009). In this case, migrating interneurons were shown to be stimulated to migrate during early phases of tangential migration while, upon increased expression of KCC2, they responded to GABA with migration arrest and generation of secondary neurites (Bortone and Polleux, 2009). More detailed experiments aimed at understanding the downstream mechanisms of GABA, during morphological maturation in pyramidal cells and interneurons, have supported the notion that upon GABA receptor activation, the subsequent depolarization triggers the activation of L-type calcium channels, which ultimately affect neurite outgrowth (Maric et al., 2001; Bortone and Polleux, 2009).

On the mechanisms of GABA, a depolarization action linked to the downstream activation of voltage-dependent calcium channels surges as a transversal effect operating during neurogenesis, migration and morphological development of all cortical neurons (Behar et al., 1996; Maric et al., 2001; Soria and Valdeolmillos, 2002; Heck et al., 2007; Bando et al., 2016). Moreover, whenever the identity of calcium channels affected by GABA-induced depolarization has been investigated, L-type calcium channels and no others have been found to be predominantly involved (Maric et al., 2001; Bortone and Polleux, 2009; Bando et al., 2016). However, beyond calcium rise, downstream events remain poorly understood. Among possible calcium targets operating during development are calcium calmodulin-dependent kinases (Takemoto-Kimura et al., 2007) and transcription of c-Fos and BDNF (Berninger et al., 1995).

### Glycine Receptors

This small amino acid neurotransmitter receptor is better known by its role in mediating synaptic transmission in the spinal cord through the activation of glycine receptors (Colin et al., 1998; Scain et al., 2010). However, alpha 2 subunit containing glycine receptors (GLRA2) are widely expressed in the developing cerebral cortex (Kuhse et al., 1991; Malosio et al., 1991; Flint et al., 1998; Avila et al., 2013b, 2014). On the other hand, in contrast to the developing spinal cord, where radial glial cells are the main source of glycine (Scain et al., 2010), in the developing cortex, glycine and taurine are both detected on immature neurons (Flint et al., 1998; Avila et al., 2013b). HPLC-based studies have quantified the neurotransmitter content in the developing cortex showing that the concentration of glycine is more than four times higher than the concentration of GABA at the embryonic day 13, while taurine concentration is even higher (Benítez-Diaz et al., 2003; Qian et al., 2014). Thus, glycine receptor-mediated signaling might have been neglected for years in favor of research on the role of GABA during brain development.

The first experiments designed to assess the functionality of cortical glycine receptors demonstrated the presence of glycine receptor-mediated currents in the cortical plate and intermediated zone, while currents were absent in the ventricular zone during late embryonic development of the rat cortex (Flint et al., 1998). However, when earlier time points were investigated, glycine elicited current was detected in new-born neurons of the ventricular zone (Noctor et al., 2004). Complementary experiments using calcium imaging as a readout of glycine receptor activation confirmed receptor functionality in the cortical plate at E17 (Young-Pearse et al., 2006) and E13 in mice (Platel et al., 2005). Importantly, calcium imaging at E13 (Platel et al., 2005) and electrophysiological recordings in the ventricular zone of the developing cortex (Noctor et al., 2004) were suggestive of a role of glycine receptors in the proliferation and differentiation of dorsal progenitors (Avila et al., 2013a). This putative role was later studied using *Glra2* knock-out mice lacking the expression of the alpha 2 subunit of glycine receptors (Avila et al., 2014). In this later study, glycine receptor expression at the ventricular zone was confirmed using immunostaining, electrophysiology and calcium imaging. Interestingly, glycine receptors were found at the subventricular zone only before E15, which may explain why earlier studies performed at later time point did not find glycine receptor expression at the proliferative zones (Flint et al., 1998). Having clarified previous discrepancies on the expression of glycine receptors, detailed morphological measurements of the developing brain of knock-out mice evidenced important anatomical defects in the brain of animals lacking *Glra2* gene. Absence of this gene was found to lead to moderated microcephaly arising from decreased number of upper layer excitatory neurons and to a lesser extent from fewer interneurons (Avila et al., 2014). Cellular mechanisms that were put forward included a direct control of the differentiation process of PAX6 positive progenitor cells. In animals lacking glycine receptors, PAX6 positive progenitors shifted from primarily generating TBR2 intermediate progenitors to directly generate lower layer cortical neurons in a higher proportion. This had the consequence of anticipately depleting the pool of progenitors at early embryonic stages compromising the generation of later born neurons such as upper layer cortical neurons. All this was supported by findings on the changed cleavage plane of PAX6 positive cells, increased cell cycle exit at E13, higher proportion of TBR1-TBR2 double-positive neurons at E12 and decreased numbers of CUX1 and CTIP2 positive neurons at birth (Avila et al., 2014). This later study is the most in deep study conducted on the role of glycine receptors on the neurogenesis process and formation of the brain and contributes with mechanistic insights on how glycine receptors control brain development.

Paralleling the advances on the role of GABA controlling cell migration and benefiting from the fact that cortical glycine receptors are almost exclusively formed by the alpha 2 subunit during cortical development, the use of the *Glra2* knock-out mice allowed to evaluate in detail the contribution of glycine receptors to the control of cell migration. As a first approach, a study conducted *in vitro*, where neurons were labeled using BrdU and glycine receptors were blocked using strychnine, suggested that glycine receptors could influence radial migration in embryonic mice (Nimmervoll et al., 2011). However, it remains to be addressed if the actions detected *in vitro* occur *in vivo*. Especially considering that the results obtained using BrdU labeling might be affected by the more recently described function of glycine receptors in controlling the neurogenic process (Avila et al., 2014). On the other hand, the role of glycine receptors in controlling the migration of cortical inhibitory neurons has become clearer. Specifically, using the *Glra2* knock-out mice and two-photon microscopy, it has been demonstrated that removal of glycine receptors impairs interneuron migration. In consequence, *Glra2* knock-out mice have a decreased number of interneurons in the cortex, which likely generates a state of hyper-excitability in young pups (Morelli et al., 2017).

In addition to the mechanisms operating downstream receptor activation described for the actions of GABA, glycine receptors have also been shown to activate voltage gated sodium channels leading to glutamate release at E13 (Platel et al., 2005). Moreover, the activation of calcium channels by glycine receptors is coupled to the phosphorylation of myosin in migratory interneurons (Avila et al., 2013b). Nevertheless, more detailed mechanisms describing the actions of glycine receptors on the control of the neurogenesis process remain to be investigated.

### Glutamate Receptors

Glutamate is the classic excitatory neurotransmitter and the main mediator of synaptic transmission in the brain. However, evolutionary studies suggest that the original function of glutamate might have been extra synaptic (Hirai et al., 2018). In neurons, glutamate can act on ionotropic iGluR, NMDA, kinate and delta receptors as well as on metabotropic receptors at synaptic and extra-synaptic sites (Herman and Jahr, 2007; Chiu and Jahr, 2017). In general, due to the variety of subunits that can form GluA receptors, it has been difficult to probe their role during development of the mammalian central nervous system (Hirai et al., 2018). Nevertheless, during development, NMDA receptors were found to be differentially expressed in the layers of the developing cortex, concentrating their expression in the cortical plate (LoTurco et al., 1991). In fact, they were found to be completely absent from ventricular progenitor cells, which only began to express NMDA receptors somewhere after their last mitotic division. In contrast, *in vivo* experiments using ketamine, an NMDA receptor antagonist, demonstrated a dose-dependent reduction in the number of BrdU-labeled cells in the developing cortex suggesting that ketamine and NMDA receptor blockage can indeed inhibit cellular proliferation in neurogenic regions (Huang et al., 2015; Dong et al., 2016). In turn, NMDA receptor blockage by ketamine would be promoting the differentiation of cortical progenitors into astrocytes during late embryonic development (Huang et al., 2015). Moreover, although progenitor cells might be insensitive to any harm, new born neurons can be dramatically affected by ketamine-induced NMDA receptor blockage (Wang et al., 2017). These results are further supported by numerous studies that show that NMDA receptors play an important role in regulating proliferation and differentiation of neuronal progenitors (Luk et al., 2003; Keilhoff et al., 2004; Kitayama et al., 2004; Joo et al., 2007; Mochizuki et al., 2007; Nácher et al., 2007). Regarding AMPA and Kinate receptors, they were found to be present at the ventricular zone of the cortex and to be particularly important in controlling the neurogenesis process by signaling to progenitors to differentiate (LoTurco et al., 1995). More detailed experiments found that while activation of these receptors increased ventricular zone proliferation, it also decreased sub-ventricular zone cell proliferation with an accompanying decrease in the generation of postmitotic neurons (Haydar et al., 2000). This differential effect was observed to be dependent on AMPA/kainate receptor activation as it was blocked using CNQX. Remarkably, in human fetal neural progenitor cells (NPCs) isolated from fetal brain tissue, it was found that AMPA and not NMDA receptor activation induces neuronal differentiation (Whitney et al., 2008). Coincidently, numerous studies have confirmed a role of AMPA/kainate receptors in the control of proliferation and differentiation of neuronal progenitors in the hippocampus (Gray and Sundstrom, 1998; Bernabeu and Sharp, 2000; Bai et al., 2003; Jiang et al., 2004; Poulsen et al., 2005).

On the effects of glutamate controlling cell migration, evidence suggested early on that glutamate was even more potent than GABA as a chemotropic for migrating principal neurons acting on NMDA receptors (Behar et al., 1999). Testing of blocking NMDA receptors showed that activation of NMDA receptor was needed for new born neurons to migrate (Komuro and Rakic, 1993; Hirai et al., 1999). Similarly, migrating interneurons were also suggested to be affected by NMDA receptor activation (Soria and Valdeolmillos, 2002; Bortone and Polleux, 2009). As expected, activation of NMDA receptor on ventricular zone derived neurons leads to a transient increase of intracellular calcium, which was linked to promotion of cell migration (Behar et al., 1999). Consequently, chelation of calcium resulted in inhibited neuronal migration (Hirai et al., 1999). Unexpectedly, NMDA agonist stimulation resulted as well in inhibition of cell migration when tested *in vitro* (Kihara et al., 2002). This is hypothesized to result from a narrow window of action in which NMDA receptor activation would be controlling cell migration. Remarkably, a recent study has analyzed the effects of NMDA receptor blockade on cell migration due to its clinical relevance (Yuryev et al., 2018). *In vivo* time-lapse imaging of the developing cerebral cortex on E14–15 embryos demonstrated that neuronal migration was strongly inhibited by the anesthetic ketamine while isoflurane had no effect. This study highlights an important consequence that may arise from the use of ketamine during pregnancy (Yuryev et al., 2018). Beyond this effect on cell migration, at this point it is important to stress that interfering with glutamate signaling during brain development has been associated with numerous neurodevelopmental disorders (Jansson and Akerman, 2014).

Non-NMDA glutamate receptors are known to be expressed in migrating cortical interneurons (Métin et al., 2000; Soria and Valdeolmillos, 2002). Specifically, migrating interneurons were shown to express calcium-permeable AMPA and kainate receptors while migrating through the intermediate zone (Métin et al., 2000; Soria and Valdeolmillos, 2002) in addition to NMDA receptors (Soria and Valdeolmillos, 2002). As expected, in this case, AMPA receptor activation was also linked to promoting cell migration since AMPA receptor blockade decreased cell motility by increasing interneuron pausing time (Bortone and Polleux, 2009). Similarly, in the case of radial migration, blocking AMPA/kainate receptors lead to enhanced extension of radial processes and reduced motility of new born neurons isolated from E14 rat embryos (Jansson et al., 2013).

### Other Lesser Studied Neurotransmitter Receptors With Neurodevelopmental Roles

Normally associated with fine tuning of superior functions of the brain, serotonin can act on several serotonin receptors. They are all coupled to G protein except for the 5HT-3 ionotropic serotonin receptor. Consequently, the effects of serotonin on the developing cerebral cortex are almost exclusively mediated by the activation of metabotropic receptors (Vitalis and Parnavelas, 2003; Riccio et al., 2009, 2011). Despite that, the 5HT-3 receptor is widely distributed in the developing nervous system along with serotonin transporters (Tecott et al., 1995) where they influence the behavior of migratory interneurons originated at the caudal ganglionic eminence (Vitalis et al., 2007; Murthy et al., 2014). Remarkably, this neurodevelopmental function comes in anticipation of a different role that has been suggested for 5HT-3 receptors to play in the adult brain, where it would be modulating interneuron activity (Morales and Bloom, 1997). This dual nature resembles the actions of GABA and glycine and seems to be a common feature of neurotransmitter-gated ion channels. Another neurontransmitter receptor, lesser studied in the context of brain development, is the nicotinic acetyl choline receptor (nAchR). This receptor is specifically activated by nicotine and thus it was studied for its potential involvement in the detrimental effects of nicotine. Specifically, it was found that nAchR activation induced apoptosis and cell death of hippocampal progenitor cells (Berger et al., 1998).

## Neurotransmission on Maturing Reprogrammed Neurons

### Lessons From Monolayer Cultures

In the last decade, the field of neuroscience has been revolutionized by an explosive advance in the experimental strategies linked to the use of human-induced pluripotent stem cells (hiPSCs). Since the first time that patients derived cell were obtained (Dimos et al., 2008; Park et al., 2008), this new technology has been widely adopted to study the effects of mutations, assess the effects of drugs, attempt cell replacement therapies and to investigate developmental biology processes in a human context. Although obtained from reprogramming of somatic cells, hiPSCs are equivalent in every aspect to embryonic stem cells keeping their remarkable ability to differentiate into any adult cell. To date, there are several methods available to differentiate hiPSCs toward specific neuronal subtypes (Bibel et al., 2007; Farra et al., 2012; Espuny-Camacho et al., 2013; Zhang et al., 2013; Kelava and Lancaster, 2016; Liu et al., 2017; Qian et al., 2018). Despite that, generating neurons directly from fibroblasts is also possible and this could be a more appropriated approach, when attempting to model conditions with an important aging component, since age-dependent transcriptomic signatures are preserved when doing so in contrast to the differentiation induced on hiPSCs, which involves the rejuvenation of cells and the restart of the developmental process (Mertens et al., 2015). Independently of the applied methodology, a series of protein markers such as nestin, b-III tubulin, MAP2 and NeuN are used to evidence a neural cell phenotype, which does not always represent a mature state in these neuronal models (Belinsky et al., 2011; Lepski et al., 2011, 2013; Maroof et al., 2013; Prè et al., 2014). In particular, the functional maturation of hiPSCs-derived neurons is better assessed attending to their electrophysiological properties (Prè et al., 2014). Thus, time of differentiation affects the maturation of action potentials (APs), generation of sizable sodium and potassium currents, evolution of passive membrane properties and appearance of spontaneous synaptic activity (Song et al., 2012; Zhang et al., 2013; Prè et al., 2014; Kang et al., 2017). Some of the first attempts to generate human neuronal models were based on the direct conversion of hiPSCs into reprogrammed neurons, but these protocols failed to recapitulate all the functional properties of mature neurons (Qiang et al., 2011; Karow et al., 2012). In turn, a more robust generation of mature electrophysiological properties, in an even shorter time of differentiation, was reached with co-culture of hiPSCs-derived neurons with glial cells (Tang et al., 2013; Zhang et al., 2013). Individually cultured hiPSCs, differentiated into mature forebrain-type neurons, showed an expected time-dependent decrease of the RMP after 48–55 days. However, evolution of this parameter was speeded up with co-culture of glial cells (Prè et al., 2014). In addition, the amplitude of the AP, the inward sodium current and the frequency of spontaneous synaptic events were enhanced even before 2 weeks of culture, when using the co-culture strategy (Tang et al., 2013; Zhang et al., 2013; Prè et al., 2014). In line with the positive effect reported when using glial cells in the differentiation of hiPSCs into neurons, a recent report evidenced that astrocyte conditioned medium (ACM) improves the functional maturation rate of neurons by hyperpolarizing the RMP and increasing their spontaneous activity (Kemp et al., 2016). A possible mechanism operating when using ACM involves the modulation of calcium currents (D’Ascenzo et al., 2006; Lepski et al., 2013; Kang et al., 2017). In particular, the pharmacological blockage of calcium channels reveals that L-type, N-type and R-type calcium channels contribute to enhance the differentiation rate when using ACM. Remarkably, the effect of ACM is also mediated by GABA_A_ during an early stage of differentiation (Kemp et al., 2016). Thereby, this data indicates that GABA neurotransmitter could promote the neuronal differentiation *via* a non-canonical mechanism of transmission.

The consolidation of functional synapses goes along with morphological changes and alterations on the gene expression pattern (Kang et al., 2017). In-depth characterization of morphology of hiPSCs-derived NPCs shows that neurite length, axon length, and the secondary and tertiary branches of dendrites were increased after 10 days of differentiation (Kang et al., 2017). With focus on the electrophysiological properties, the molecular components that determine the morphological changes of hiPSCs during differentiation into neurons have been studied by using pharmacological inhibition of ROCK GTPases (Harbom et al., 2019). However, while ROCK inhibitor accelerated the generation of complex morphology, it did not contribute to the development of mature electrophysiological activity (Harbom et al., 2019). In contrast, the phosphodiesterase inhibitor 3-Isobutyl-1-methylxanthine (IBMX) was shown to increase the level of cAMP and to promote the mature morphology after 1 week of differentiation accelerating the generation of APs and increasing the amplitude of sodium, potassium and L-type calcium currents (Lepski et al., 2013). On the other hand, AP-evoked vesicular release underlies the molecular assembly of presynaptic release machinery at the CNS (Südhof, 2013). This process depends on the homeostasis and maintenance of a myriad of synaptic proteins whose signaling mechanisms have not been broadly described. Nevertheless, in essence, synaptic communication relies on regulated secretion through two pathways: synaptic vesicles (SVs) and dense-core vesicles (DCVs; Rizo and Südhof, 2012; Persoon et al., 2018). In this context, co-culture of differentiated hiPSCs-derived GABAergic neurons with glial cells evidenced both, SV and DCV markers. Moreover, calcium/SNARE dependency was observed at 50 days of *in vitro* culture and while DCVs showed microtubule-depending anterograde transport, which was markedly faster in axons than dendrites, dendrites showed similar anterograde and retrograde transport. Interestingly, while DCV secretion increased until 36 days in culture, SV secretion steadily increased for up to 50 days (Emperador Melero et al., 2017).

### Lessons From Cultured Organoids

The “Fit-for-purpose” concept in the bioengineering field points out that, for example, brain organ-like structures are a model system able to provide complementary insights on cellular aspects such as morphogenesis, regionalization of specific structures, and the spatial array of circuits (arealization), as well as cell fate and specification (Jabaudon and Lancaster, 2018). Thus, cultured organoids are not expected to necessarily be able to address all developmental biology issues. Moreover, experimental reproducibility is still an important issue to take into account when drawing conclusion from data collected at different laboratories using organoids (Lancaster et al., 2013; Qian et al., 2016, 2018, 2019; Mansour et al., 2018; Giandomenico et al., 2019; Singh et al., 2019). Thus, at present, the efforts have focused on improving neural survival and the reliability of this type of culture (Eremeev et al., 2019; Giandomenico et al., 2019; Yakoub, 2019). However, guiding the development of organoids by supplementation of specific factors towards a particular lineage, or using the unguided methods relying on the intrinsic morpho-functional program and cues, offer distinct advantages to be considered (Lancaster et al., 2017; Sloan et al., 2018; Cederquist et al., 2019; Qian et al., 2019). For example, unguided methods present clear advantages to study the heterogeneity of neural progenitor subpopulations formed during the development of the brain cortex as well as for the study of the participation of neurotransmitters and their receptors during early stages of the process (Qian et al., 2019). On the other hand, recent modifications to a guided method using an air-liquid interface for the culture of organoid slices showed improved survival, enhanced self-organization, and better recapitulation *in vitro* of morphological and functional characteristics (Giandomenico et al., 2019). Despite reported differences between guided and unguided methods, they both are similar in the sense that both methods allow cells to undergo an intrinsic developmental program that mimic molecular (Camp et al., 2015; Quadrato et al., 2017; Amiri et al., 2018), cellular (Kadoshima et al., 2014; Qian et al., 2016; Bagley et al., 2017; Birey et al., 2017; Velasco et al., 2019) and physiological (Paşca et al., 2015; Qian et al., 2016; Birey et al., 2017) characteristics of a wide spectrum of conditions being powerful biological systems to move forward in the comprehension of early human brain cortex development (Kadoshima et al., 2014; Camp et al., 2015; Qian et al., 2016; Bershteyn et al., 2017; Birey et al., 2017; Arlotta and Paşca, 2019; Velasco et al., 2019). Remarkably, brain organoids are also beginning to be used to study synaptic transmission and the effects of neurotransmitters. Moreover, recent evidence shows that similar electrophysiological properties can be detected in both differentiated bi-dimensional and three-dimensional cultures (Chandrasekaran et al., 2017). Along this line, optimized cerebral organoids were shown to have increased abundance of neurotransmitter receptors such as glutamate, AMPA receptor GluA1, NMDA receptor GluN1, GluN2A and GluN2B, and GABA-B receptor 1 at the mRNA and protein expression levels (Yakoub, 2019). Moreover, experiments on slices obtained from organoids showed that organoid-derived neurons have the capability of firing TTX-sensitive APs during stimulation as well as displaying prominent Na^+^–K^+^ currents in response to voltage ramps (Qian et al., 2016). These results are all consistent with a previous report using human cortical spheroids (hCSs), in which similar electrophysiological events were recorded (Paşca et al., 2015). Also, in the latter two studies, astrocytes were found by functional recording analysis, highlighting another important feature of the method applied for the generation of their organoids (Paşca et al., 2015; Qian et al., 2016). Even more importantly, half percent of the neural population showed sEPSC (Paşca et al., 2015; Qian et al., 2016). Despite eventual discrepancies, the development of functional parameters, in organoids, progresses according to the expected neural maturation and the accompanying depolarizing-hyperpolarizing GABA switch evidenced by the reduced abundance of NKCC1 cotransporter and the enrichment of KCC2 symporter; this last one is strongly expressed in the cortical plate (CP) after 80 days of differentiation. Regarding patterns of calcium activity, while immature organoids display calcium rise in response to GABA, more mature ones respond in a higher proportion exclusively to glutamate (Qian et al., 2016). Similar findings were observed in dissociated and entire hCSs loaded with Fura-2 and Fluo-4 probes, respectively (Lancaster et al., 2013; Paşca et al., 2015). A more refined approach, using the overexpression of GCaMP6s calcium sensor and the Na^+^ channel blocker TTX, indicated that calcium oscillation in organoids can be used as readout of neuronal activity, as in more mature embryonic structures (Xiang et al., 2017). Likewise, pharmacological application of glutamate increased the frequency of calcium spikes in the organoid system (Lancaster et al., 2013). Also, using specific spheroids to model cortical neurons forming the pallium (glutamatergic neurons) and subpallium (GABAergic neurons), it was found that neurosteroids and allopregnanolone (an agonist of GABA_A_ receptor) in combination with retinoic acid can increase the frequency of spontaneous calcium spikes as well (Birey et al., 2017). Taken together, these results indicate that functional neurons and neural connections are effectively established in cultured organoids and they have the ability to generate complex synaptic networks. This is more evident after long periods of culture, where cortical superior layer type neurons are born with characteristic molecular traits and functional dendritic spines (Quadrato et al., 2017; Giandomenico et al., 2019; Trujillo et al., 2019). Importantly, genetic and morphological signatures are consistent with human prefrontal cortex development (Zhong et al., 2018). Functional measurements of network activity in organoids have been performed using close-packed silicon microelectrode technology (multi-electrode array, MEA), which has offered an overview of synaptic connectivity and, in particular, it has served to measure aggregates of synaptic currents as local field potentials (LFP) helping to determine various characteristics of the generated networks. As expected, under TTX treatment, recorded AP and spontaneous neural firing were abolished (Quadrato et al., 2017; Trujillo et al., 2019). This result is also consistent with MEA analysis performed in air-liquid interface grown cerebral organoids (Giandomenico et al., 2019). More in detail, mathematical cross-correlation of recorded spike trains on monosynaptic connections revealed that a high number of recorded spikes (81%) resulted from synaptic transmission non-mediated by NMDA receptor (Quadrato et al., 2017). Oscillatory network dynamic in cortical organoids has more recently been evaluated using MEA data analysis as well (Trujillo et al., 2019). In this regard, pharmacological modulation aimed at blocking glutamatergic activity and enhancing GABAergic synaptic transmission, resulted in a depressed synaptic response evidenced in the reduction of electrical activity and the loss of synchronicity (Trujillo et al., 2019). Hence, recorded calcium dynamic was also affected after pharmacological treatment with the glutamate receptor antagonist CNQX (Sakaguchi et al., 2019) while inhibition of GABAergic transmission increased the number of synchronized electrical responses (Trujillo et al., 2019). Thus, these results show that cultured organoids can display synchronous network activity resembling human cortical network development creating opportunities to study early actions of cellular neurotransmission.

## Modeling Synaptic Dysfunctions and Mental Disorders Using Human Reprogrammed Neurons

### The Use of hiPSCs to Enlighten the Etiology of Neurodevelopmental Disorders and Autism

In humans, the development of the brain is a sequential process orchestrated by dynamic patterns of gene expression and various intrinsic and extrinsic signaling molecules (Cardoso et al., 2019; Silva et al., 2019). This process start at the third week of embryonic development when the neuroectoderm folds to give shape to the neural tube (Stiles and Jernigan, 2010; Tau and Peterson, 2010). Underlying this process is intense cell proliferation, migration, differentiation and synaptogenesis. Thus, there are multiple cellular mechanisms that could be impaired and lead to impaired brain development, which also depends on sensitive and critical timeframes where brain development is more vulnerable (Meredith, 2015; Bennett et al., 2019). Modeling a condition involving neurodevelopmental disorders is a complex endeavor because the general nature of such disorders is heterogeneous and its approach requires the efficient characterization of the distinct cellular subtypes and circuits affected in correlation with the hallmark phenotypes of the pathology. Commonly, the clinical manifestations in patients occur at birth or during infancy showing alterations in several brain functions such as learning, memory, cognition and sensorimotor behavior (Korkmaz, 2011). In this context, studies using hiPSCs, in the context of the role of shank 2 and 3 and the etiology of autism spectrum disorder (ASD), supported the participation of synaptic proteins as key factors for neurodevelopmental disorders (Shcheglovitov et al., 2013; Yi et al., 2016; Vitrac and Cloëz-Tayarani, 2018; Zaslavsky et al., 2019).

We are recently starting to unravel the way of how genetic variants act in ASD patients. While many genetic factors and the environment contribute to the diversity of ASD (Prilutsky et al., 2014; Cardoso et al., 2019), the regulation of synaptic structure and function emerged as a relevant aspect linked to the etiology of ASD. Analysis of autistic patients with functional magnetic resonance imaging (fMRI) revealed reduced neuronal connectivity in brain areas related to social interaction whereas stronger neuronal connectivity was found in areas associated with repetitive behavior (Monk et al., 2009; Habela et al., 2016). Consistently, hiPSCs-derived cortical-like neurons from patients with idiopathic autism displayed reduced frequency of excitatory postsynaptic currents (EPSCs) with altered kinetics. Additionally, electrophysiological recording showed a decrease in voltage-gated sodium and fast potassium currents. Interestingly, the morphological analysis of reprogrammed neurons indicated that the number of neurites were similar to control, even though the density of synaptic protein markers was moderately reduced (Liu et al., 2017). Remarkably, the analysis of gene expression evidenced that synaptic pathways-related genes can also be increased, specially, the expression of GABA_A_ receptor alpha 3 (GABRA3), and voltage-gated sodium type II alpha subunit (SCN2A; Mariani et al., 2015; Liu et al., 2017). In this context, with the use of organoids, it was evidenced that increased expression of GABA_A_ exert an imbalance between glutamatergic and GABAergic neuronal fate. The overproduction of GABAergic neurons is attributable to an early increase of FOXOG1 expression, a protein that promotes the proliferation of GABAergic precursors and acquisition of GABAergic neuronal fate (Mariani et al., 2015). Furthermore, the GABAergic neuron overproduction increased GABAergic synapses with an exuberant cellular overgrowth of neurites, which was also displayed at the transcriptome analysis. In this regard, a comparative RNA-Seq study between the transcriptional pattern of gene expression of hiPSCs-derived cortical neurons from patients with idiopathic ASD and controls after 35 and 135 days after induction showed a dysregulation of genes involved in neuronal differentiation, axon guidance, cell migration, metabolism of nucleic acid, and neural region patterning (Mariani et al., 2015; DeRosa et al., 2018). In the same study, more specific findings revealed a significant decrease in the number of spontaneous calcium transients, a readout of neuronal activity and developmental expression of AMPA and NMDA receptors. Also, scratch assays showed a significantly decreased extension of neuronal processes in ASD hiPSCs-derived neurons after 35 days of induction (Kruszewski, 2003; Brembeck et al., 2006; DeRosa et al., 2018). Thus, the analysis of the interaction between various altered mechanisms in a temporal context can contribute to a better understanding of neurodevelopmental disorders. In this line, a recent attempt to find a critical developmental period integrating cellular states and molecular determinants showed that transcriptional networks that produce aberrant neuronal maturation are regulated by gene modules which appeared to be accelerated in ASD NSCs (Schafer et al., 2019). Surprisingly, bypassing the NSCs stage by direct conversion of NSCs into cortical neurons prevented the display of neuronal ASD characteristics. These results are directly related to the intrinsic developmental changes in timing, named heterochronicity, presented during development. In connection to this particular aspect, organoids obtained from ASD patients evidenced a thinning cortical plate with a shift on migrating TBR1 positive neurons (Schafer et al., 2019). These results support the idea of a defective intrinsic transcriptional program in ASD cortical neuron development (Schafer et al., 2019). On the other hand, ASD has been related with Kleefstra syndrome (KS-ASD), a rare genetic disorder characterized by developmental delay, intellectual disability and anatomical abnormalities. KS-ASD is caused by the haploinsufficiency of the euchromatic histone lysinemethyltransferase 1 (*EHMT1*) gene that catalyzes the mono- and di-methylation at lys-9 position of histone H3, suggesting possible roles in neurodevelopment. Cortical neurons derived from KS-ASD patients showed reduced expression of *EHMT1* mRNA and protein, and displayed shorter neurites as well as reduced arborization and dendritic protrusion density. In line with the defective morphology, calcium responses to acetylcholine stimuli evidenced reduced nicotinic cholinergic tone after 5 weeks of culture. Furthermore, low expression of migration and maturation genes was found. Thus, KS-ASD could be framed in the ASD umbrella and hiPSC-derived neurons could serve as another model to improve the comprehension of these mental disorders (Nagy et al., 2017).

Cultured brain organoids are able to mimic with high fidelity the spatial distribution of neural progenitors, their expansion process and their differentiation into mature neurons (Kadoshima et al., 2014; Qian et al., 2016, 2019; Birey et al., 2017; Quadrato et al., 2017; Renner et al., 2017; Xiang et al., 2017). In this line, autism is an archetypical disorder for the use of three-dimensional models because genetic alterations are often linked to the regulation of neural development leading to excitatory-inhibitory imbalance in the cortex (Mariani et al., 2015; Fatehullah et al., 2016; Nageshappa et al., 2016; Nestor et al., 2016; Choi et al., 2017). More in detail, an early study using an organoid-based three-dimensional model for ASD showed accelerated cell cycle and increased production of GABAergic interneurons (Mariani et al., 2015). Additionally, a recent report showed that in periventricular heterotopia (PH), a common disorder characterized by neurons that do not migrate from their sites of production outward to form the cerebral cortex, organoids recapitulate the loss of structural organization of germinal zones in patients with mutations or absence of protocadherins DCHS1 and FAT4 (Klaus et al., 2019). As a consequence of this condition, increased neural nodules were observed at the equivalent ventricular zones (Klaus et al., 2019). On the other hand, modeling prenatal hypoxic injury (HI) with cultured organoids showed that transient hypoxia activated and prolonged apoptosis leading to the loss of specific outer radial glia progenitor cells (Daviaud et al., 2019). Thus, organoid models are beginning to provide important new insights in the cellular mechanisms of neurodevelopmental disorders and neurodegenerative diseases (Qian et al., 2016, 2018; Choi et al., 2017; Gabriel and Gopalakrishnan, 2017; Lee et al., 2017; Shah and Singh, 2017; Chen et al., 2019; Klaus et al., 2019; Paşca, 2019; Setia and Muotri, 2019).

### hiPSC and Rett Syndrome

Rett syndrome (RTT) is one of the most severe genetic neurodevelopment disorders affecting females. The hallmarks of this disease include a normal development up to 6–18 months of age and a later deceleration of head growth and microcephaly during the RTT onset. The disorder as well is accompanied by growth retardation, weight loss and muscle hypotonia. Additionally, the onset of the syndrome is followed by impairments in cognition, social abilities and motor function (Chahrour and Zoghbi, 2007). RTT is caused in over 90% of cases by mutations in the *MECP2* gene, but another minor fraction includes mutations in either *FOXG1* and *CDKL5* genes (Amir et al., 1999; Chahrour and Zoghbi, 2007; Amenduni et al., 2011; Guerrini and Parrini, 2012). In this context, hiPSCs-derived neurons from RTT patients expressing mutated MECP2 showed a reduced pallidin mRNA transcript. Pallidin participates in the protein interaction network of dysbindin and it is required for the biogenesis of lysosome-related organelles complex 1 (BLOC-1), which was suggested to be altered in autism and schizophrenia (Larimore et al., 2013). Initial studies made use of fibroblasts from young girls expressing mutated *MECP2* gene. They were used to assess and later establish the ability to reprogram somatic cells observing positive expression of βIII-Tubulin and Nestin in RTT-hiPSCs after differentiation (Hotta et al., 2009; Ananiev et al., 2011). However, analysis of fluorescence intensity indicated a reduced signal against βIII-Tubulin marker and sodium channels in RTT-hiPSCs-derived neurons compared to controls (Kim et al., 2011). This observation could be a consequence of small soma size (Cheung et al., 2011). In a successful way, the generation of *in vitro* neuronal models from RTT hiPSCs containing MECP2 mutations evidenced a reduced number of dendritic spines (Marchetto et al., 2010). In addition, the analysis of calcium transients in selected hiPSCs-derived neurons, whose synaptic activity response could be blocked with TTX or the glutamate receptor antagonists, showed a decreased frequency and amplitude of calcium events evidencing impaired connectivity (Marchetto et al., 2010). Likewise, these models presented defects to generate stable APs, suggesting a sodium channel impairment related to a diminished peak inward sodium currents (Farra et al., 2012). Moreover, sEPSC were reduced in amplitude compared to controls (Marchetto et al., 2010). Thus, these findings confirmed an altered neuronal network in hiPSCs-derived neurons from RTT patients. More recently, it has been demonstrated an altered KCC2 in RTT syndrome hiPSCs-derived neurons. MECP2 protein regulates KCC2 expression inhibiting the RE1-silencing transcription factor, a neuronal gene repressor. This results in lower expression of KCC2 and a delayed functional switch of GABA from excitation to inhibition evidenced by GABA-evoked currents under various holding potentials (Tang et al., 2016). Interestingly, the overexpression of KCC2 or IGF-1 treatments restored the functional switch of GABA suggesting that KCC2 could be a therapeutic target to relieve RTT.

### Advances on Understanding Ethanol Effects Using Human Reprogrammed Neurons

Recently, research on extrinsic factors affecting brain development has also been facilitated by the adoption of methods based on human reprogrammed neurons. In this context, ethanol research has also been facilitated by the use of hiPSCs-derived neurons. In these cells, transcriptome studies have shown that several notch signaling pathway genes that regulate cell fate and synaptic plasticity are affected by alcohol exposure (Jensen et al., 2019). Furthermore, genes for NMDA and GABA_A_ subunits show significantly elevated expression on human iPSCs-derived forebrain neurons after chronic treatment with 50 mM alcohol for 7 and 21 days, respectively with daily changes (Lieberman et al., 2012, 2018). However, while electrophysiological analyses did show that GABA_A_- and AMPA-evoked current responses were unaffected in hiPSCs-derived neurons, a reduced amplitude of NMDA response after acute exposure to ethanol was reported (Lieberman et al., 2012). On the other hand, studies linked to the effect of ethanol as primary cause of depletion of the stem cell pool either in adult neurogenesis or prenatal development have been described (Vangipuram and Lyman, 2010; Le Maître et al., 2018). Even though a high cytotoxic concentration of ethanol could be responsible for its effect in the developing brain, these findings suggest that ethanol could impair early cellular processes in the developing CNS.

## Perspectives

### Neurodevelopmental Function of Neurotransmitters in Light of Recent Progress Using Reprogrammed Neurons

As it has been presented, neurotransmitters play relevant roles not only during synaptic transmission and plasticity but also ensuring the correct execution of the developmental program of the brain. Importantly, nowadays it is possible to track down the participation of neurotransmitters in the processes that originate human neuronal circuits by taking advantage of the reprogramming and differentiation of somatic cells. In this context, it was shown that manipulating the action of GABA during differentiation had a dramatic effect on the rate of neurogenic cell divisions giving rise to a homogeneous neuronal progeny (Kemp et al., 2016). This is striking because it implies that modulation of neurotransmitter systems can in principle be used to guide the differentiation process *in vitro* and contribute to the generation of drug screening assays. On the other hand, potential effects of interfering with neurotransmitter systems can be better assessed in reprogrammed neurons informing about the human teratogenic potential of a certain drug. This notion is further supported by studies on the effect of a NMDA receptor blocker. In particular, ketamine deleterious effects have been investigated using hiPSCs-derived neurons showing how new born neurons can be dramatically affected by ketamine-induced NMDA receptor blockage (Wang et al., 2017).

### Using Reprogrammed Neurons for Modeling Neurodevelopmental Disorders

Early adoption of the use of reprogrammed neurons for the modeling of neurodevelopmental disorders leads to great success in recapitulating some of the most important characteristics of the disorder of interest (Espuny-Camacho et al., 2013; Lancaster et al., 2013). In fact, the use of iPSC-derived neurons in research conducted to understand the involvement of synaptic dysfunctions in the etiology of autism has already inspired new therapeutic interventions for neurodevelopmental disorders (Darville et al., 2016). However, it has been evidenced that the methodology needs to be advanced in order to reduce the experimental variability or else only the most extreme phenotypes of the disease could be reproduced (Sandoe and Eggan, 2013; Brennand et al., 2015). More recently, reprogramming methods have been standardized to the point where variability is truly convening the inherent variability found on individuals from where the cells were obtained (Kilpinen et al., 2017). Nevertheless, approaches have varied when attempting differentiating hiPSCs into neurons (Dimos et al., 2008; Espuny-Camacho et al., 2013; Lancaster et al., 2013; Kemp et al., 2016). In this particular, our research on the role of neurotransmitters during cerebral cortical development has made us wonder how some important developmental signals and events are not being considered when attempting to model a disease phenotype. For instance, manipulating GABA signaling was shown to dramatically speed up the differentiation process directing it to a synchronized more mature inhibitory phenotype (Kemp et al., 2016). Moreover, in the process there was a synchronized maturation of neuronal progenitors which differs substantially from the timely controlled generation of each cortical cell type occurring during normal corticogenesis. Along the same line, commercially available differentiation media and reagents have been optimized in order to decrease the culture time needed to obtain mature neuronal activity bypassing normal developmental processes and manipulating the concentration of neurotransmitters (Bardy et al., 2015). As a consequence, while improved differentiation protocols can help to understand human-specific mechanisms of disease, they can also be the source of important experimental artifacts.

## Author Contributions

JO and AÁ both equally contributed to the conception, design, drafting and revising of the work. Consequently, JO and AÁ provided approval for publication of the content and agree to be accountable for all aspects of the work.

## Conflict of Interest

The authors declare that the research was conducted in the absence of any commercial or financial relationships that could be construed as a potential conflict of interest.
